# A call for increased transparency and accountability of health care outcomes in US Immigration and Customs Enforcement detention centers

**DOI:** 10.1016/j.lana.2024.100825

**Published:** 2024-06-24

**Authors:** Annette M. Dekker, Amy Zeidan, Joseph Nwadiuko, Elizabeth Jordan, Parveen Parmar

**Affiliations:** a Department of Emergency Medicine, David Geffen School of Medicine, University of California, Los Angeles, USA; b Department of Emergency Medicine, Emory School of Medicine, Emory University, USA; c Department of Health Policy and Management, Fielding School of Public Health, University of California, Los Angeles, USA; d Sturm College of Law, University of Denver, USA; e Department of Emergency Medicine, Harbor UCLA Medical Center, University of California, Los Angeles, USA

**Keywords:** Health policy, Immigration, Detention, Monitoring

## Abstract

Concerns over health care in US Immigration and Customs Enforcement (ICE) facilities have grown over the past decade, including reports of medical mismanagement, inadequate mental health care, and inappropriate use of solitary confinement. Despite being a federally funded agency, reporting and accountability of health outcomes in ICE facilities is limited. This manuscript outlines current standards for health in ICE detention, how compliance is evaluated, why this process fails, and how current processes can be improved to achieve transparency and accountability. Ultimately, health metrics must be: 1) frequent; 2) timely; 3) granular; 4) collected by an independent body; and 5) publicly reported. Financial compensation for health service providers must be contingent on meeting these required metrics, with contract termination for persistent violations. Transparent and accountable monitoring systems, as are required in other federally funded healthcare facilities, are essential to accurately measure health outcomes and harms of individuals held in detention.

## Introduction

Over the last 30 years, the use of immigration detention has increased significantly in the United States, with nearly 38,000 individuals detained in over 100 Immigration and Customs Enforcement (ICE) facilities on a given day in 2024, with an average length of stay of 48 days.^1^ Concurrently, human rights organizations have reported concerns over medical care in detention facilities,^2^ which has been substantiated by evidence in the medical literature. Studies evaluating deaths in ICE detention facilities reveal systematic failures and violations of ICE's own standards, leading to potentially avoidable deaths among individuals with few pre-existing conditions.^3^^,^^4^ Grossly inadequate mental health care has been cited with an 11-fold increase in rate of suicide deaths in detention centers over the past decade^5^ and excessive use of solitary confinement, despite studies documenting increased mental health harm–including suicidality–resulting from this practice.^6^ Finally, formerly detained individuals describe neglect of basic human needs, including harmful living conditions, insufficient access to health care, and new conditions arising as a result of detention.^7^

In the absence of abolition of immigration detention centers, there is an urgent need to improve the reporting, transparency, and accountability of health standards in detention centers to accurately measure health outcomes and harms. Below we outline current standards for health in ICE detention, how compliance is evaluated, why this process fails, and how current processes may be improved to achieve transparency and accountability. Given scope limitations, this paper will not address conditions in Customs and Border Protection facilities, which are intended to screen and house individuals upon arrival to the US for less than 72 hours prior to transfer to ICE facilities or release. The authors note that standards and accountability mechanisms are similar, if not worse, in these facilities and require future attention.^8^^,^^9^

## Current health standards, reporting, and accountability in ICE detention facilities

### Standards

ICE is a federally funded agency under the US Department of Homeland Security (DHS). Within ICE, the ICE Health Service Corps (IHSC) is responsible for medical care. IHSC provides both direct medical care as well as oversight of private corporations that provide medical care in non-IHSC staffed facilities, operating a budget of approximately $323.7 million in 2022.^10^

Health care standards in ICE detention facilities are dictated by either the National Detention Standards or Performance-Based National Detention Standards (PBNDS) depending on the facility type and year of initiation.^11^ The standards share many similarities; however, the National Detention Standards typically apply to contracts with local government facilities that do not exclusively house immigrants or facilities that have an average daily population of less than 10, whereas PBNDS typically applies to facilities that exclusively house detained immigrants and are often run by private corporations. Currently over 80 percent of individuals held in detention are in facilities that must comply with the 2016 revised PBNDS.^1^ As such, we will focus on these standards in more detail.

The 2016 revised PBNDS include standards addressing: medical, mental health, and dental screening; routine and preventive health care; emergency care; specialty care; timely responses to medical concerns; hospitalization; and professional language services for individuals with limited English proficiency.^12^ Additionally, standards require sufficient and appropriately-trained medical staff, including 24/7 mental health staffing. Despite an extensive list of priorities, the standards outlined in PBNDS are often vague and lack specific guidelines that facilities must adhere to. Furthermore, although PBNDS requires that facilities enact onsite monitoring of health service outcomes with plans to address concerns, there is no instruction on what health metrics should be monitored nor what outcomes would be considered concerning.

### PBNDS audits and publicly reported health metrics

Since October 2022, ICE has relied on its Office of Detention Oversight (ODO) to conduct annual facility inspections to audit compliance with PBNDS (Table 1).^13^ Inspections are scheduled in advance, allowing facilities to temporarily modify conditions to ensure a passing inspection.^18^ Moreover, while each facility is inspected annually, ODO does not audit all standards annually. Rather, ODO rotates the standards it inspects such that all standards are assessed at least once over a three-year period.^13^ The publicly available ODO inspections report compliance with each standard as present or deficit. No additional information is provided to support how decisions were made or what deficiencies were found, limiting the ability of independent entities to monitor health of individuals in detention. Furthermore, no timeframe is provided for follow-up inspections to assess whether deficiencies were addressed.

**Table 1:** Publicly reported metrics on individuals in ICE detention.

Agency	Name	Date of Reports	Metrics *Italics indicate Health Specific*	Timeliness	Granularity	Limitations
ICE [reports to DHS]	Detention Statistics^1^	2019—present	ADP, by criminality and threat level	YTD; updated monthly	By facility	Does not include demographics or any health outcomes. Prior data available annual rather than monthly.
			ALOS			
			Segregation, by type	Quarterly	Aggregate across all facilities	
ODO^a^ [reports to ICE]	Facility Inspection^13^	2011–2022	Compliance with PBNDS	Every 3 years	By facility	Minimal detail why standards were met or not. Standards evaluated every 3 years rather than annually.
		2022—present	Compliance with PBNDS	Annually (standards rotated on 3-year basis)		
OIDO [reports to DHS]	Facility Inspection^14^	2022—present	Compliance with PBNDS	Variable	By facility	Infrequent and unpredictable.
OIG [reports to DHS]	Audits, Inspections, and Evaluations^15^	2003—present	Variable	Variable	Aggregate across all facilities and by facility	Infrequent and unpredictable.
ICE [reports to DHS]	Death review^16^	2018—present	*Events of death*	Within 90 days of death	By individual	Limited details of medical data related to death.
Nakamoto Group Inc^a^ [reports to ICE]	Facility Inspection^17^	2018–2022	Compliance with PBNDS	Annually	By facility	No longer active. Limited explanation of how data collected or defined. No quality metrics.
			*Death by cause*			
			Segregation by type			
			*Detainees in medical observation*	Monthly		
			*Detainees in mental health observation*			
			*Infectious diseases reported/confirmed*			
			*Outside medical referrals*			
			*Detainees transported to off-site hospital for emergency care*			
			*Admissions to off-site hospitals for medical reasons*			
			*Admissions to off-site hospitals for mental health reasons*			
			*Sick call requests [requests for medical attention]*			
			*Sick call encounters [encounters for medical attention]*			
			*Suicide watches*			
			*Suicide attempt*			
			*Hunger strikes*			
			*Grievances, by type and outcome*			
			*Physical assault*			
			Disciplinary action, by outcome			
			Special housing, by reason			
			Use of force, by type			
			*Sexual assault, by type and outcome*			

ADP: Average daily population.

ALOS: Average length of stay.

DHS: Department of Homeland Security.

ICE: Immigration and Customs Enforcement.

ODO: Office of Detention Oversight.

OIDO: Office of the Immigration Detention Ombudsman.

OIG: Office of Inspector General.

YTD: Fiscal year to date.

a Contractual responsibility for ICE Enforcement and Removal Operations facility inspections transferred from Nakamoto Group Inc to ODO on October 1, 2022.

Prior to October 2022, the Nakamoto Group Inc. was contracted to inspect facilities annually to determine compliance with select detention standards, while ODO also conducted inspections in parallel every three years.^17^ Although the Nakamoto publicly available reports included health metrics no longer included in the current ODO-led facility inspection reports, such as the number of medical emergencies, infectious diseases, and suicide attempts, Nakamoto inspections were found to be ‘significantly limited’ and ‘inadequate’ by DHS oversight bodies.^18^

Aside from ODO facility inspection reports, additional publicly reported health metrics in ICE detention centers are sparse (Table 1). Those that are released are reported with variability and include special reports and death reviews. Special reports are conducted by the Office of Inspector General (OIG) and Office of the Immigration Detention Ombudsman (OIDO). Unlike ODO, these agencies report directly to DHS rather than ICE, allowing for greater independence in reporting.^15^^,^^14^ These reports generally investigate either a specific facility or broad concerns impacting multiple facilities. While these reports often provide the most detailed accounts of health conditions, the topics and regularity of reports are sporadic and unpredictable. Finally, any death that occurs for an individual held in ICE custody results in a publicly reported account of the events leading to the death that is released within 90 days.^16^ Details in these reports are limited compared to internal reviews not publicly released.

### Accountability

Under the 2009 DHS Appropriations Act, facilities that fail two consecutive performance evaluations will lose their contracts with ICE.^19^ No repercussions exist for a singular inspection failure. DHS oversight bodies such as OIG have raised concerns that facility inspections do not hold facilities with poor conditions accountable. First, reported deficiencies are assumed to be less than actual conditions given that inspections are announced, infrequent, and limited in scope.^18^ In addition, OIG has found that ICE inappropriately uses waivers to allow facilities to opt out of compliance with certain standards, such as posting emergency plans.^18^ Second, repeated deficiencies are common, and often persist without repercussions. For example, in 2015, ODO identified 18 repeat deficiencies during 23 inspections, and in 2016, ODO identified 21 repeat deficiencies in 29 inspections.^18^ A report conducted by the US Government Accountability Office found that repeat deficiencies do not result in facility closure or financial penalties.^20^ As one ICE employee stated, inspections are “very, very, very difficult to fail”.^18^ Third, OIG has found that inspections that evaluate standards every three years are “too infrequent to ensure the facilities implement all corrections,” allowing dangerous conditions to exist for years without consequence.^18^ The result is a system in which deficiencies in health care provision are ignored or overlooked and, when identified, rarely result in accountability or improvement of conditions.

## A case example: failure of existing facility inspections to address access to and care standard deficiencies at Stewart Detention Center in Lumpkin, Georgia

Seven deaths occurred at Stewart Detention Center from 2018 to 2022.^16^ Yet, publicly reported Nakamoto inspections of Stewart Detention Center found no medical deficiencies in 2018, 2019, 2020, 2021, or 2022.^17^ In 2019, ODO inspections found five medical deficiencies including delays in comprehensive medical evaluations upon arrival to the facility.^13^ Repeat ODO inspections in 2020, 2021, and 2022 do not mention whether these deficiencies were re-evaluated or addressed; no new medical deficiencies are noted in subsequent years.^13^ In November 2022, OIG made an unannounced inspection of Stewart Detention Center. The inspection found several deficiencies in medical staff responses to detainee requests for medical attention, such that only fifty percent of requests were addressed, and even then, those who were seen experienced long delays.^21^

Four of the seven deaths at Stewart Detention Center included individuals that required hospitalization within the first sixteen days of arrival to the detention center, raising the question of whether a more comprehensive evaluation on arrival could have prevented their death given ODO's notation of intake deficiencies in 2019.^16^ Furthermore, five of the deaths were due to infectious causes that occurred both prior to and during the COVID-19 pandemic, bringing into question whether delays for medical evaluation—as noted by OIG—contributed to mortality. While ICE has publicly stated that it has addressed these deficiencies, to date, no independent agency has verified improvements. Since the OIG report has been published, two additional individuals at the detention center have died.^16^^,^^22^

## Alternative health metrics used by health services researchers

Health services researchers have utilized several alternative strategies to obtain data about healthcare in ICE facilities. One strategy has been through submission of Freedom of Information Act requests, which are often done in partnership with legal experts. Evidence of systematic substandard care and inappropriate use of solitary confinement have largely been understood as a result of Freedom of Information Act requests.^3^^,^^6^^,^^23^ These requests, however, are notoriously slow, often requiring litigation, and even then, records are routinely incomplete.^6^

Health services researchers have also studied medical records for care individuals receive outside of detention facilities as proxies to understanding care within detention centers, including data from emergency medical services^24^ and hospitalizations.^25^ Some states, including California, have instituted their own oversight mechanisms, which have allowed for contextualization of health care in detention facilities.^24^^,^^26^ Most states, however, do not have such a mechanism. Without transparency of protocols, resources, and existing health needs within detention centers, researchers are limited in their ability to contextualize findings from outside medical records.

Finally, understanding of health outcomes has also come from interviews with individuals previously detained in ICE facilities,^7^^,^^27^ however this approach is unable to provide a voice to those currently in detention and limited by fears about documentation status.

## A call for increased reporting, transparency, and accountability

Transparency and accountability in health systems are essential to protect the health of individuals receiving care. Fully independent, high-quality monitoring systems that are publicly reported and directly tied to financial compensation are not only accepted standards of care, but they serve as safeguards for individuals’ health and wellbeing.

For example, the majority of hospital systems in the United States receive compensation for Medicare through the Centers for Medicare and Medicaid Services. Unlike ICE facility inspections, hospitals lose accreditation by the Centers for Medicare and Medicaid Services—and associated financial compensation—if identified deficiencies are not resolved within six months, or if there is an immediate threat to safety.^28^ This exists in direct contrast to a recent investigation of an ICE facility, where an inspector found several examples of negligence, noting “any of these findings alone can be considered an ‘Immediate Jeopardy’ according to the Centers for Medicare and Medicaid Services and can lead to the closure of large health systems”.^23^ The facility remains open.

Unlike PBNDS metrics under ICE, the Centers for Medicare and Medicaid Services metrics are publicly available and additional financial incentives exist for achieving certain quality metrics. These metrics are specific and actionable, ranging from management of chronic disease (i.e. percentage of patients with diabetes with hemoglobin A1c less than 9%) to provision of mental health care (i.e. percentage of patients with follow up with a mental health provider within 7 days of a hospitalization for mental illness).^29^ Furthermore, data usage agreements that allow for sharing of deidentified, individual-level data that follow individuals across multiple events in publicly funded health systems, such as Medicaid, are commonplace.^30^ In comparison, ICE exists as an anomaly in its drastic lack of quality monitoring and accountability of health care outcomes.^8^^,^^26^

### Proposed metrics

We call for increased publicly reported health care metrics and accountability in ICE detention facilities consistent with recommendations put forth by the World Health Organization, United Nations High Commissioner for Refugees, and US immigration organizations.^31^, ^32^, ^33^ Unlike current reported measures, suggested metrics should be: 1) frequent, i.e. monthly; 2) timely, i.e. within 30 days of event; 3) granular, i.e. on facility level; 4) collected by an independent body; and 5) publicly reported (Table 2). Metrics should include facility characteristics, demographics of individuals detained, and process and quality metrics as described in other federally funded health systems such as the Centers for Medicare and Medicaid Services.^29^

**Table 2:** Call for increased publicly reported health metrics.

Examples of Recommended Reported Health Metrics
**General Principles of Reporting**
• Publicly available • Frequent (i.e. monthly) • Timely (i.e. within 30 days of event) • Granular (i.e. on facility level, or when appropriate, individual level) • Independent (i.e. gathered by objective outside agency)
**Facility Characteristics**
• Facility capacity and average daily population • Number of medical staff by specialty, level of training, licensing status, basic/advanced cardiovascular life support certification • Staffing ratios, including number of individuals responsible for as well as number of medical oversight • Facility protocols including activation of 911, 24-h emergency care plans, onsite medical care, onsite psychiatric care, care for vulnerable populations, quality assurance, offsite referrals, and discharge
**Individual Characteristics**^a^
• Gender, age, race, primary language spoken, county of origin • Number of individuals with medical conditions, including communicable disease, non-communicable disease, severe mental illness • Number of individuals in vulnerable groups, including LGBTQIA+^b^, disabilities, pregnant, victim of prior assault/trauma
**Process Metrics**
• Number of requests for medical evaluations, by illness/injury • Number of medical evaluations, by illness/injury • Number of individuals held in medical units, by illness/injury, average length of time • Number of individuals held in solitary confinement, by reason (administrative/disciplinary), medical, average length of time • Number of individuals with medical emergencies, by illness/injury • Number of individuals hospitalized, by illness/injury • Number of medical related complaints
**Quality Metrics**
Access to Care
• Number of individuals offered preventative screening (i.e. pap smear) • Length of time between admission and medical screening, initiation of appropriate medications, and mental health evaluation • Training level of health care provider seen for health care request • Length of time from request for health care/mental health to being seen by health care provider • Length of time from placement of referral for specialty/mental health care to time of appointment • Number of referrals missed, reason why referral missed • Access to HIV medications (% missed medication, with reason) • Access to psychiatric medication (% missed medication, with reason)
Health Outcomes
• Hemoglobin A1c for individuals with diabetes • Blood pressure for individuals with hypertension • Number of suicide attempts with comprehensive review • Number of deaths with comprehensive review (including individuals released within 30 days)

a Reportable when the aggregate number is sufficiently large such that individual identities are protected, otherwise recommend reporting on regional level.

b Lesbian, gay, bisexual, transgender, queer, intersex, or asexual.

Publicly reported facility characteristics should include basic data such as capacity and average daily population, as well as the number of medical staff and their appropriate qualifications. Facilities should also report the staffing ratios, oversight, and which providers are onsite. Facilities must also provide protocols for medical care, including activation of 911, onsite medical care, onsite psychiatric care, offsite referrals, care for vulnerable populations, and internal quality monitoring and assurance. In addition to facility characteristics, basic demographics of individuals detained should be reported such as age, gender, and race, as well as number of those with specific medical conditions and members of vulnerable groups.

Publicly reported metrics should include process and quality metrics. Examples of process metrics include requests for medical attention, offsite emergencies, and hospitalizations. Quality metrics should include both access to care and health outcomes. Access to care metrics should measure delays in care, including access to chronic disease management, acute illness, emergency care, specialty care, and mental health care. Access to care metrics should also capture whether individuals are seen by the appropriate level of provider. Health outcome metrics should include metrics for specific diseases as well as comprehensive review of suicide attempts and deaths. Finally, deidentified, individual-level data should be shared when possible, such that individuals could be monitored across multiple health events.

The metrics discussed here would be improvements to current health monitoring but are by no means inclusive of all the needed changes to current standards. Rather, these metrics provide a starting framework that can be expanded based on input from research, policy, and government stakeholders. Furthermore, it must be reiterated that these metrics are of limited value if they are not publicly reported on a regular and timely basis on a facility level. Accuracy of these metrics must be ensured by an independent agency given previously discussed concerns of validity of data.

Finally, we strongly emphasize the need for accountability for deficiencies in health standards and outcomes. Financial compensation for health service providers must be contingent on meeting the metrics described, with contract termination for repeated violations, as is commonplace in other federally funded health systems. With a publicly funded budget of over $300 million, IHSC and private-contracted health care providers within ICE detention must be held accountable to the standards set forth in their contracts. Feasibility of implementing such metrics and accountability has been shown in other publicly funded federal health systems. The most significant barrier to implementation is ICE's willingness and commitment to issue a systemwide policy directive and re-negotiate contracts with private corporations, or alternatively, the ability of legislators to pass relevant policy in an already polarized political climate.

Given the ongoing documented harms of ICE detention and inadequate existing health monitoring and accountability, the safest immediate response would be ending ICE detention. However, with no immediate end in sight, transparent and accountable quality monitoring systems are essential to accurately measure health outcomes and harms of individuals held in detention.

## Contributions

Concept and design: AMD, AZ, JN, EJ, PP.

Draft of the manuscript: AMD, AZ.

Critical review and revision of the manuscript for important intellectual content: AMD, AZ, JN, EJ, PP.

## Declaration of interests

The authors declare no competing interests.
